# Effects of breathing a hyperoxic gas mixture on perceptual, biochemical and performance recovery following simulated soccer match play

**DOI:** 10.5114/biolsport.2025.146785

**Published:** 2025-01-16

**Authors:** Wael Daab, Haithem Rebai, Abd-Elbasset Abaïdia, Mohamed Amine Bouzid

**Affiliations:** 1 College of Sport Science, University of Kalba, Sharjah, United Arab Emirates; 2 Research Laboratory Sports Performance Optimization (LR09SEP01), National Center of Medicine and Science in Sports (CNMSS), Tunis, Tunisia; 3 UR UPJV 3300 APERE Adaptation Physiologiques à l’Exercice et Réadaptation à l’Effort, Université de Picardie Jules Verne, Amiens, France; 4 Research Laboratory: Education, Motricité, Sport et Santé, EM2S, LR19JS01, High Institute of Sport and Physical Education, University of Sfax, Tunisia

**Keywords:** Hyperoxia, Fatigue, Stress, Hooper index, Recovery, Soccer, Football

## Abstract

The aim of this study was to examine the effect of breathing a hyperoxic gas mixture on recovery kinetics after a simulated soccer match protocol. In a double-blind, randomized design, twenty-eight semi-professional soccer players completed the Loughborough Intermittent Shuttle Test (LIST) followed by the administration of either a hyperoxic (FIO_2_ = 99.5%: HYP) or a normoxic gas mixture (FIO_2_ = 21%: NORM). HYP and NORM were administered immediately after LIST and daily for the next 3 days for 15 minutes. Physical performance (squat jump: SJ, countermovement jump: CMJ, maximal voluntary contraction: MVC, and 20 m sprint: SP), blood concentrations of muscle damage markers (creatine kinase: CK, lactate dehydrogenase: LDH), marker of inflammation (C-reactive protein: CRP) and Hooper index (HI) were assessed at baseline, 15 minutes and 24 h, 48 h and 72 h following the LIST. SJ, CMJ, MVC, and SP were lower at 15 min, 24 h, and 48 h in both conditions compared to the prior LIST (p < 0.05). However, the decrease in MVC was significantly attenuated at 15 min, 24 h and 48 h in HYP compared to the NORM condition (p < 0.01). Likewise, HI, muscle soreness and fatigue scores were significantly lower in HYP compared to the NORM condition up to 48 h following the LIST (p < 0.01). The present study suggests that the application of HYP immediately after a simulated soccer match and for the next 3 days promoted a lower fatigue-induced decrement in MVC and restored perceptual parameters of fatigue in semi-professional players.

## INTRODUCTION

It is well documented that breathing oxygen-enriched air (hyperoxia) during continuous exercise enhances performance [1]. However, the use of hyperoxia after high-intensity exercise has received limited scientific attention, and conflicting results have been reported regarding the effectiveness of this practice in restoring metabolic homeostasis [2] or accelerating recovery following exercise-induced muscle damage [3]. Additionally, the efficacy of using hyperoxia to promote the recovery processes remains questionable [4]. Researchers and practitioners are constantly seeking ways to accelerate recovery, and the breathing of hyperoxic gas has been a relatively under-researched recovery modality for athletes. Yet, most of the research in this field has been conducted in unhealthy populations, such as those suffering from chronic obstructive pulmonary disease (COPD). It has been shown that the use of hyperoxia in these populations during the recovery period after exercise accelerates the recovery time from dynamic hyperinflation. [5]. When trialled in healthy athletic populations, the use of hyperoxia provided during the recovery periods between interval repetitions was shown to speed up the rate of haemoglobin restoration [2]. However, no effect has been shown on post-exercise blood lactate clearance rates, perceptual recovery between exercise repetitions, or performance during subsequent bouts of exercise [6].

Soccer is a strenuous contact team sport characterized by high-intensity intermittent activities [7]. During high-intensity exercise, alterations in O_2_ delivery to the working muscles could influence the development of fatigue, likely by increasing the rate of metabolites, which will eventually decrease muscle pH and/or by disturbing the ionic balance across the muscle membrane [8]. Furthermore, a soccer match involves many eccentric contraction movements including changes of direction, tackles, accelerations, and decelerations [9]. Consequently, exposure to eccentric contraction may cause a marked inflammatory response and impair physical performance [10].

The processes of muscle repair and regeneration are complex and involve cellular and molecular events such as muscle vascularization, innervations and the recovery of the contractile properties of the muscle [11]. Interestingly, increasing the amount of oxygen in the muscle could enhance the removal of the metabolites [11, 12] and accelerate the processes of muscle regeneration and repair [11]. In this regard, it has been shown that satellite cells play an important role in the process of muscle repair and regeneration [13]. Following damaging exercise, these cells help to repair the muscle by fusing with existing fibres or creating new fibres.

Previous studies showed that breathing a hyperoxic gas (99.5% O_2_ for 20 minutes) improved the perception of recovery after three swimming sessions in highly trained athletes [14]. In addition, HYP restored maximal voluntary contraction (MVC) following intermittent intense exercise [15]. However, the efficiency of hyperoxia to accelerate the recovery process in soccer players has not been investigated. Recently, research by Mihailovic et al. [16] showed that hyperbaric oxygenation using a hyperbaric chamber enhances the recovery of performance and perceptual parameters following fatiguing exercise. However, such a strategy is difficult to implement in a soccer context as it requires advanced equipment. From a practical point of view, the use of hyperoxia by breathing a gas immediately after a soccer match would be an attractive approach to improve the recovery kinetics as it is a low-cost solution.

To the best of our knowledge, no study has investigated the effects of HYP following a simulated soccer protocol able to induce similar responses to a typical soccer competition. Therefore, the present study examined the effect of breathing a hyperoxic gas (99.5% O_2_) on recovery kinetics of physical, biochemical, and perceptual parameters after a simulated match play protocol in soccer players. We hypothesized that HYP would accelerate the recovery following an intermittent running protocol that simulates soccer.

## MATERIALS AND METHODS

### Participants

Twenty-eight male soccer players volunteered to participate (mean ± SD: age: 24.5 ± 3.6, height = 183 ± 1.3 cm, body mass = 79.3 ± 2.3 kg, estimated maximal oxygen uptake (${\dot{\text{V}}\text{O}}_{2\max}$) 55.5 ± 3.2 ml kg^−1^ min^−1^) (Table 1). All participants, members of the same squad playing in the second division of the local National League, were trained between 4 and 5 days per week for ~60 to 70 min per session and played one official match every week. The present investigation was conducted in accordance with the Declaration of Helsinki and approved by the local clinical Research Ethics Committee (CPP N° 0104). The sample size was calculated a priori using software (G*Power version 3.1), according to the recommendations of Beck (2013) [17], to reduce the probability of type II error and to determine the minimum number of participants required for this investigation. The criteria used for calculating the sample size were as follows: an effect size (f) of 0.25, significance level (α) of 0.05, and a desired statistical power (1-β) of 0.80. Considering the study design with two groups and five measurement time points, a correlation among repeated measures of 0.5, and assuming sphericity (ε = 1), the minimal required sample size calculated in the power analysis was 20 participants.

**TABLE 1:** Descriptive characteristics and baseline values of participants in the NORM and HYP conditions.

	NORM condition	HYP condition
**Age (years)**	24.5 ± 3.6	–
**Weight (kg)**	79.7 ± 2.3	79.83 ± 2.55
**Height (cm)**	183 ± 1.3	–
**Body mass index (kg/m^2^)**	23.8 ± 1.7	–
**Body Fat (%)**	9.89 ± 2.45	9.92 ± 2.98
**Total body water (%)**	66.18 ± 1.26	66.08 ± 1.34
**Average Heart rate (bpm)**	169 ± 10	168 ± 11
**Maximal Oxygen Uptake (${\dot{\text{V}}\text{O}}_{2\max}$) ml · kg^−1^ · min^−1^**	55.5 ± 3.2	–
**Maximal voluntary contraction (N)**	909 ± 108	911 ± 175

### Experimental design

**FIG. 1:**
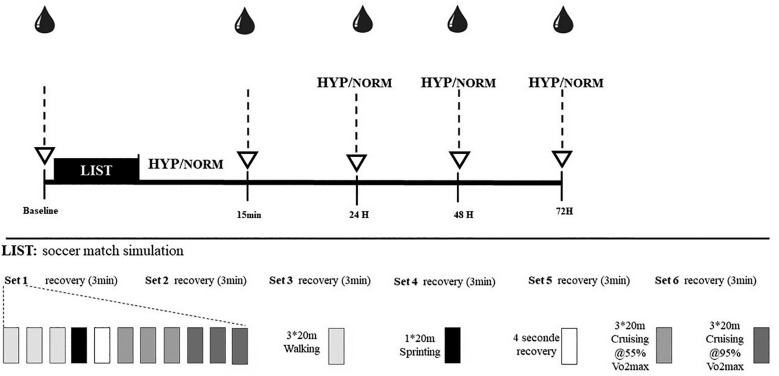
Overview of the entire experimental period. Drop marks denote the time points when blood samples were taken. Downward arrows denote the time points when physical performance tests and perceptual ratings were recorded.

A schematic representation of the experimental design is shown in Figure 1. All participants completed two preliminary visits to ensure familiarization with the measurements, and another session to evaluate ${\dot{\text{V}}\text{O}}_{2\max}$ using the 20 m shuttle run test [18]. Afterwards, subjects completed two experimental sessions separated by 14 days in which they performed a simulated soccer match followed by 15 min of recovery during which period they were breathing either a hyperoxic gas mixture with a fraction of inspired oxygen (FIO_2_) of 99.5% O_2_ (HYP) or a normoxic gas mixture with an FIO_2_ of 21% (NORM). Physical performance and biochemical and perceptual parameters were assessed at baseline, 15 min (immediately after the gas breathing), 24, 48 and 72 h after the LIST. The order of testing was the same in both HYP and NORM; firstly, the subjective ratings were recorded, followed by the biochemical parameters, followed by the physical performance tests. Subjects were asked to avoid the use of any recovery method, any practice of training or physical activity, to not consume alcohol, protein or caffeine one day prior to each LIST and three days after. Carbohydrate and protein intake was adjusted to each player’s body weight to meet the recommendations for daily recovery, in order to limit the influence of dietary intake on test results. This study was a double-blind, randomized design. Players were randomly assigned to either the HYP (n = 14) or the NORM (n = 14) condition. Finally, all assessments took place in the late off-season to the early pre-season phase of the players’ training year and were performed on an indoor hall under standardized conditions (temperature: 21–23°C; relative humidity: 40–60%) at the same time of the day.

### Recovery intervention

In a double-blind order and immediately, 24 h, 48 h and 72 h following the LIST, both gas treatments were provided for the participants via a high concentration facemask with the reservoir connected to a medical gas cylinder. The gas was delivered for the entire 15 min recovery period. During HYP treatment, the administration of gas was regulated by an oxygen regulator and a 15 L flow meter (Oxygen Flowmeter, Medical gas Installers Group, US). The flow rate was 15 L/min for the hyperoxia session (HYP) and 1 L/min for the NORM session.

### Simulated soccer match

The LIST protocol required participants to perform 20-m shuttle runs at various intensities [19]. The movement pattern during the test was as follows:

– 3 × 20 m at walking pace
– 1 × 20 m at maximal running speed
– 4 seconds recovery
– 3 × 20 m at a running speed corresponding to 55% of individual ${\dot{\text{V}}\text{O}}_{2\max}$
– 3 × 20 m at a running speed corresponding to 95% of individual ${\dot{\text{V}}\text{O}}_{2\max}$

The test consisted of six exercise sets approximately 15 min long for each set, separated by periods of 3 min rest (Figure 1). Players were informed about their running and walking speed every 20 m using audible signals issued by software developed for this purpose. Heart rate was continuously recorded throughout the LIST using heart rate monitors (Polar Team System, Polar electro, Kempele, Finland).

### Perceptual scales

The Hooper index (HI) questionnaire was collected every morning at the same time of the day before the LIST, and at 15 min, 24 h, 48 h and 72 h after it. HI has been considered as useful and sensitive tool for monitoring fatigue and perceptual status in soccer players [20]. It has four main elements: fatigue, sleep, stress and muscle pain. Each part is described on a 1–7 points scale, where 1 represents “very, very good” and “7” represents “very, very poor” wellness ratings. Hence, low scores indicate better recovery. Finally, the total HI score was computed by summing its four parts [21].

### Blood collection and biochemical analysis

Blood biomarker concentration was determined from the venous blood samples collected into an EDTA tube before each session. Whole blood was centrifuged at 1500 rpm for 15 min at 4 °C, and then processed for plasma and stored at −70 °C until analysed. C-reactive protein (CRP) was analysed through an immunoturbidimetric assay. Intra-assay and inter-assay coefficients of variation (CV) for CRP were 2.3% and 3.1, respectively. Moreover, test kits (A11A01632, Horiba-ABX, Montpellier, France; myoglobin bioMerieux 30446) were used to determine the concentration of creatine kinase (CK) and lactate dehydrogenase (LDH). Intra-and inter-assay coefficients of variation were 3.4% and 5.3% for CK, 2.8% and 4.5%, respectively, for LDH.

### Performance tests

The order of physical performance tests throughout the protocol was as follows: countermovement jump (CMJ), squat jump (SJ), maximal voluntary contraction of quadriceps (MVC). CMJ and SJ highest scores (Opto-jump system Microgate SARL, Italy) were used to assess muscle power.

The Opto-jump system is a widely validated tool for assessing vertical jump performance, demonstrating excellent validity and reliability in measuring flight time and jump height [22]. During CMJ and SJ, participants were instructed to keep their hands on their hips throughout the movement and squat down to approximately 90° knee flexion. Three attempts were given with a 60 second rest between with the best score recorded.

MVC was determined using an isometric dynamometer (Good Strength, Metitur, Finland), which has demonstrated reliability and validity in measuring isometric force [23]. After performing a set of three sub-maximal repetitions of knee extension at the referred angle as a warm-up, players were asked to push and hold the contraction for 3 seconds. Three attempts were performed, each separated by 2 minutes of passive recovery, and the best attempt was recorded for the analysis.

For the sprinting performance (SP), players were asked to start each sprint from a line placed 50 cm before the starting line. Upon a verbal signal, they ran as fast as possible for 20 m. Time was registered using a photocell timing system (Brower Timing System, IRDT175, Draper, UT, USA) placed at 0 and 20 m. Players completed two runs separated by a 1-minute recovery period, with the fastest time recorded for analysis.

### Statistical analyses

Statistical analysis was completed using Statistica for Windows software (StatSoft, version 12, Paris, France). All data are represented as mean ± standard deviation (SD). Exploratory data analysis was performed to check normality of each dependent variable using the Shapiro-Wilk test and homogeneity of variances using the Levene test. For all physical, biochemical, and perceptual data, a two-way ANOVA with repeated measures was used [Condition (HYP or NORM) × Time (baseline, 15 min, 24 h, 48 h, and 72 h after exercise)]. When appropriate, post hoc comparisons were made with the Bonferroni test. Mauchly’s test was used to test the assumption of sphericity, and if this assumption was violated, the Greenhouse-Geisser correction was applied. Effect sizes were reported as Cohen’s d and calculated to determine the magnitude of changes. Cohen’s d values of 0.2–0.59 were considered ‘small’, values of 0.6–1.19 were considered ‘medium’, and values of 1.2–2.0 were considered ‘large’ [24].

## RESULTS

### Perceptual scales

Statistical analysis revealed a significant interaction (condition × time) for HI (F_4,44_ = 6.15, p < 0.01). HI scores were higher immediately after the LIST and at 24 h and at 48 h in both conditions (p < 0.01). However, HI score was lower immediately (p < 0.01; *d* = 1.8) and at 24 h (p < 0.01; *d* = 1.6) after the LIST in the HYP compared to the NORM condition (p < 0.01) (Table 2).

**TABLE 2:** Hooper index (HI), muscle soreness, fatigue, sleep and stress scores recorded during normoxia session (NORM) and hyperoxia session (HYP)

Outcome	Condition	Baseline	15 min	24 h	48 h	72 h
HI (AU)	HYP	9.18 ± 0.18	14.36 ± 1.37*#	13.45 ± 0.86*#	13.63 ± 0.72*#	8.90 ± 0.66
	NORM	9.09 ± 0.28	17.09 ± 0.59*	15.9 ± 0.69*	15.2 ± 0.94*	9.54 ± 0.67

Fatigue (AU)	HYP	1.90 ± 0.53	3.36 ± 0.80*#	2.90 ± 0.88*#	2.81 ± 0.75*#	2 ± 0.77
	NORM	2.09 ± 0.53	4.63 ± 0.92 *	4.90 ± 0.70 *	3.54 ± 1.29 *	2.36 ± 0.92

Muscle soreness (AU)	HYP	2.95 ± 0.75	4.09 ± 1.86*	3.72 ± 0.90*	3.90 ± 0.94*	2 ± 0.63
	NORM	2.81 ± 0.60	4.72 ± 0.90*	4.81 ± 0.87*	4.18 ± 1.16*	2.09 ± 0.70

Sleep (AU)	HYP	1.90 ± 0.83	3.45 ± 1.29 *	3.36 ± 0.92 *	3.31 ± 0.80 *	2.27 ± 0.77
	NORM	2.18 ± 0.75	3 ± 0.77 *	3.54 ± 0.82 *	3.27 ± 0.64 *	2.45 ± 0.93

Stress (AU)	HYP	2.11 ± 0.77	4 ± 0.88*	3 ± 0.98*	2.9 ± 0.83*	2.63 ± 1.11
	NORM	2.21 ± 0.77	4.72 ± 1.1*	3.18 ± 0.87*	3 ± 1.09*	2.63 ± 1.12

* : Significant difference in comparison to baseline (p < 0.05).

# : Significant difference in comparison to NORM (p < 0.05).

Likewise, a significant interaction effect (condition × time) was observed for fatigue score (F_4,44_ = 7.49, p < 0.01). The fatigue score increased immediately after the LIST and at 24 h and 48 h (p < 0.01) after it in both conditions (Table 2). However, the fatigue score was lower immediately after (p < 0.01; *d* = 1.9), at 24 h (p < 0.01; d = 1.7) and 48 h (p < 0.01; *d* = 1.2) after the LIST in the HYP compared to the NORM condition (Table 2).

Stress and sleep scores increased in both conditions immediately after the LIST and at 24 h and at 48 h (P < 0.01) following it (Table 2). However, no significant difference was observed between conditions (p = 0.08).

Finally, regarding the muscle soreness score, we observed a significant interaction effect (F_4,44_ = 6.49, p < 0.01). The muscle soreness score increased immediately after the LIST and at 24 h, 48 h and at 72 h (p < 0.01) following it in both conditions. Muscle soreness scores were significantly lower at 15 min (p < 0.01; *d* = 1.6) and 24 h (p < 0.01; *d* = 1.8) after the LIST in the HYP compared to the NORM condition (P < 0.01) (Table 2).

### Biochemical parameters

**FIG. 2:**
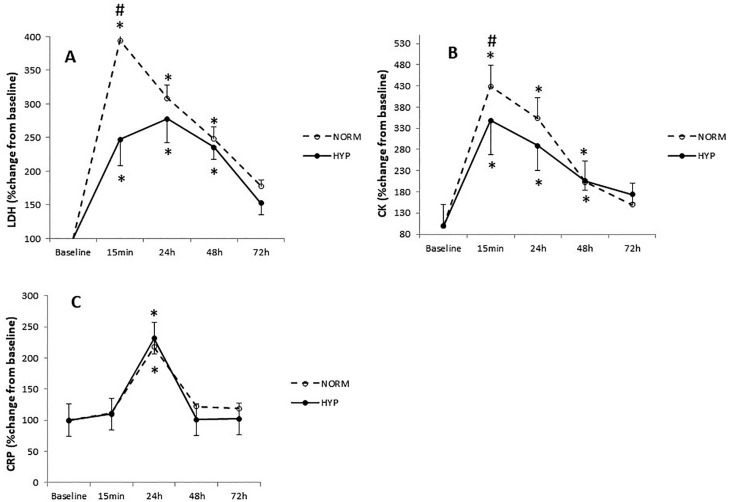
Lactate dehydrogenase (LDH) (A), plasma creatine kinase (CK) (B), C-reactive protein (CRP)(C) values recorded at baseline and following the LIST (at 15 min, 24 h, 48 h and 72 h) during normoxia session (NORM) and hyperoxia session (HYP). Values are means and standard deviations. Data were normalized as a percentage of baseline. *: Significant difference in comparison to baseline (p < 0.05). #: Significant difference in comparison to NORM (p < 0.05).

We observed a significant interaction effect for LDH activity (F_4,44_ = 6.44, p < 0.01). LDH activity increased at 15 min, 24 h, 48 h and at 72 h (p < 0.01) after the LIST in both conditions (Table 3). However, LDH activity at 15 min was higher in NORM compared to HYP (p < 0.01; *d* = 0.8) (Fig. 2A).

**TABLE 3:** Maximal Voluntary Contraction (MVC), Countermovement Jump (CMJ), Squat Jump (SJ), 20 m Sprint (SP) values and Creatine Kinase (CK), Lactate Dehydrogenase, C-reactive protein (CRP) concentration recorded during normoxia session (NORM) and hyperoxia session (HYP).

Outcome	Condition	Baseline	15 min	24 h	48 h	72 h
MVC (N)	HYP	911 ± 175	816 ± 136*#	774.2 ± 118*#	835 ± 149*#	868 ± 138
	NORM	909 ± 108	711 ± 126*	633 ± 147*	747 ± 108*	854 ± 101

CMJ (cm)	HYP	31.6 ± 2.2	30 ± 3.5*	28.7 ± 3.5*	29.3 ± 3.3*	30.5 ± 2.7
	NORM	31.7 ± 2.2	28.8 ± 3.8*	29.1 ± 3.5*	28.7 ± 3.2*	30.9 ± 2.2

SJ (cm)	HYP	30.3 ± 2.7	27.8 ± 3.1	27.5 ± 3.7*	28.1 ± 3.3	29.7 ± 2.4
	NORM	30.3 ± 2.8	29.8 ± 4.3	28.5 ± 4.2*	29.4 ± 2.7	30.3 ± 3.4

SP (S)	HYP	3.2 ± 0.1	3.3 ± 0.2*	3.4 ± 0.2*	3.5 ± 0.2*	3.3 ± 0.3
	NORM	3.2 ± 0.2	3.3 ± 0.1*	3.3 ± 0.2*	3.6 ± 0.4*	3.3 ± 0.2

CK (U/L)	HYP	199.3 ± 36	691.3 ± 186*	608.8 ± 208*	416.3 ± 135*	235.2 ± 91
	NORM	193.5 ± 74	788.3 ± 245*#	654.6 ± 187 *	327.7 ± 131*	287.5 ± 93

LDH (U/L)	HYP	151.6 ± 34	433.7 ± 52*#	438.9 ± 51*	388.2 ± 77*	256.2 ± 47
	NORM	160.9 ± 36	562 ± 198*	449.8 ± 40*	327.4 ± 38*	241.7 ± 62

CRP (mg/L)	HYP	1.51 ± 1.28	1.60 ± 1.30	3.46 ± 2.59*	1.49 ± 1.18	1.50 ± 1.20
	NORM	1.44 ± 1.31	1.58 ± 1.25	3.15 ± 2.31*	1.78 ± 1.33	1.61 ± 1.13

* : Significant difference in comparison to baseline (p < 0.05).

# : Significant difference in comparison to NORM (p < 0.05.

Statistical analysis showed a significant interaction effect for CK (F_4,44_ = 7.34, p < 0.01). CK activity increased in NORM and HYP conditions at 0 h, 24 h and 48 h (p < 0.01) (Table 3). However, CK activity at 15 min was higher in NORM compared to HYP (p < 0.01; *d* = 1.5) (Fig. 2B).

Regarding CRP activity, statistical analysis showed only a significant main of time effect (F_4,40_ = 6.83, p < 0.01). CRP activity increased only at 24 h (p < 0.01) after the LIST in both conditions without a significant difference (p = 0.08) (Fig. 2C).

### Physical performance

Statistical analysis showed an interaction between condition and time for MVC (F_4,44_ = 7.58, p < 0.01). Compared to baseline, MVC performance decreased at 15 min (−18 ± 3%, p < 0.01 in NORM; −11 ± 4%, p < 0.01 in HYP) at 24 h (−28 ± 4%, p < 0.01 in NORM; −19 ± 4%, p < 0.01) and at 48 h (−15 ± 4%, p < 0.01 in NORM; −8 ± 4%, p < 0.01 in HYP) (Table 3).

**FIG. 3:**
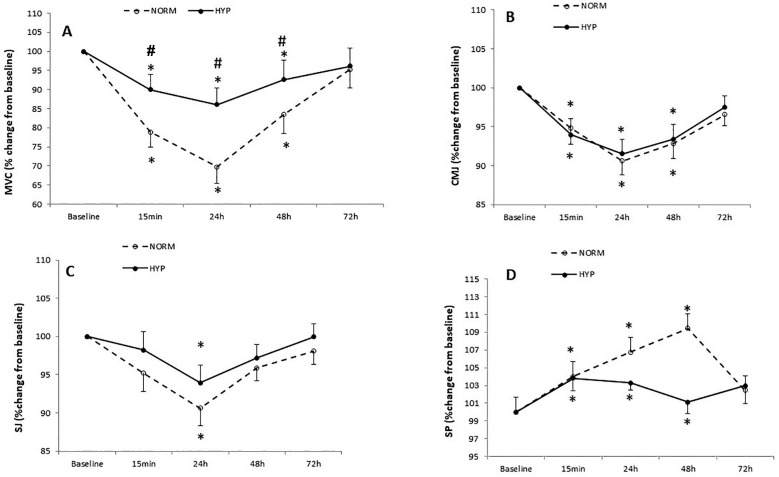
Maximal voluntary contraction of quadriceps (MVC) (A), countermovement jump (CMJ) (B), squat jump (SJ) (C) and 20 m sprint test (SP) (D) values recorded at baseline and following the intermittent test (at 15 min, 24 h, 48 h and 72 h) during normoxia session (NORM) and hyperoxia session (HYP). Values are means and standard deviations. Data were normalized as a percentage of baseline. *: Significant difference in comparison to baseline (p < 0.05). #: Significant difference in comparison to NORM (p < 0.05).

MVC values were higher at 15 min (p < 0.01; *d* = 1.3), 24 h (p < 0.01; *d* = 1.4) and 48 h (p < 0.01; *d* = 1.2) in HYP compared to NORM treatment after the LIST (Fig. 3A).

We observed a significant main effect of time in CMJ performance (F_4,44_ = 4.54, p < 0.01). Compared to baseline CMJ performance decreased at 15 min (−5 ± 2%, p = 0.03 in NORM; −6 ± 2%, p = 0.04 in HYP) at 24 h (−9 ± 2%, p = 0.02 in NORM; −8 ± 2%, p = 0.03 in HYP) and at 48 h (−7 ± 2%, p = 0.03 in NORM; −6 ± 2%, p = 0.04 in HYP) after the LIST (Table 3). No significant difference was found between NORM and HYP sessions in CMJ throughout the recovery period (p > 0.05) (Fig. 3B).

Statistical analysis showed a significant main effect of time in SJ performance (F_4,40_ = 4.83, p < 0.01). Compared to baseline, SJ decreased at 24 h (−10 ± 2%, p = 0.03 in NORM; −7 ± 2%, p = 0.04 in HYP) after the LIST (Table 3). However, no significant difference was found between the two conditions in SJ performance throughout the recovery period (p = 0.09) (Fig. 3C).

A significant interaction effect (condition × time) was observed for SP (F_4,44_ = 8.38, p < 0.01). SP performance in NORM and HYP conditions decreased at 0 h, at 24 h and at 48 h (p < 0.01) (Table 3). No significant difference was found between NORM and HYP sessions throughout the recovery period (p = 0.07) (Fig. 3D).

## DISCUSSION

The main finding of the present study is that the administration of a hyperoxic gas with 99.5% oxygen concentration for 15 minutes immediately after the LIST and every day for 3 days after it promoted a lower decrement in MVC, improved perceptual parameters and had a trivial effect on biomarkers of muscle damage through the recovery period of 72 hours.

The present study showed a significant decline in physical performance following the LIST protocol and throughout the recovery period. This finding is in accordance with previous data we have reported [11]. MVC is considered one of the most reliable and accurate tools to quantify muscle fatigue [25]. In the present study, the MVC decline at 15 min (−18 ± 3% in NORM; −11 ± 4%, in HYP) at 24 h (−28 ± 4% in NORM; −19 ± 4%) and at 48 h (−15 ± 4% in NORM; −8 ± 4% in HYP) is consistent with previous studies [9, 26]. In fact, the high amount of eccentric contractions during the LIST protocol would induce considerable damage in type II muscle fibres and consequently lead to an impairment in determinant movement in a soccer match such as jumping, sprinting and the ability of muscle to generate force [26]. However, when comparing the two conditions in our study, the repeated exposure to HYP was found more effective in attenuating the decline in MVC across the recovery period compared to NORM. These results are in line with the findings of Yokoi et al. [15], who observed that the elevation of oxygen availability by hyperoxic breathing accelerated the recovery of MVC after intermittent isometric quadriceps exercise. However, our results disagree with those reported by Harrison et al. [27], who found no effect of HYP (100% of O_2_) on isometric strength recovery following eccentric exercise. Along the same line, Abaïdia et al. [28] noted that increasing muscle oxygenation had no effect on hamstring force recovery following muscle-damaging exercise. To our knowledge, our study is the first to determine the ergogenic effects of HYP on the recovery of MVC in soccer players following simulated soccer match play.

Given the complexity of MVC, involving both peripheral and central factors, it is challenging to speculate on the potential mechanism(s) responsible for the observed improvement with HYP in our study. Oxygen supply has been suggested to enhance strength recovery after muscle injury by accelerating satellite cell proliferation and muscle fibre regeneration [27]. The role of satellite cells is to repair the muscle following damage by fusion with existing fibres or creating new fibres [11]. Chaillou & Lanner [13], in an in vivo study, found that the availability of oxygen influences satellite cells’ activity during the skeletal muscle regeneration process. Based on this evidence, we could hypothesize that increasing the availability of oxygen in the muscle with HYP would have accelerated the regeneration of damaged muscle fibres, and this would have resulted in better maintenance of muscle strength during the recovery period in our study.

Another potential mechanism could be via better motor unit recruitment following HYP. Tucker et al. [1] found that the muscle electromyographic activity was greater in HYP as compared to NORM during cycling exercise, suggesting that improved exercise performance in the HYP condition could be the result of increased capacity for the activation of motor units, which allow an increase in muscle activation. In our study, HYP was administered immediately following the LIST, a fact which could have elevated total arterial oxygen content and thus the rate of oxygen diffusion from the plasma into the muscle tissue cells. This, in turn, would have influenced the muscle function in a positive manner. As a support to this hypothesis, Verges et al. [29] showed that, when breathing a hyperoxic gas for 15 minutes, the arterial oxygen partial pressure significantly increased (up to approximately 350 mm Hg) compared with the value of the control trial (approximately 120 mm Hg), whereas arterial oxygen saturation did not change.

Increasing the rate of oxygen diffusion into muscle tissues with HYP has been associated with several positive effects, including faster phosphocreatine (PCr) resynthesis, lower glucose-6-phosphate accumulation, reduced hydrogen ion concentration (and a subsequent drop in pH), and decreased inorganic phosphate levels. [4]. All these changes with HYP could have contributed to a faster recovery compared to NORM in our study.

In agreement with previous studies [9, 30], the biochemical markers peaked immediately following the LIST and decreased at 48 h in both conditions, indicating a high level of muscle damage and inflammation after the LIST. In the present study, HYP attenuated the increases of CK and LDH at 15 min; however, no significant differences were observed between the two conditions at 24 h, 48 h and 72 h.

HI, fatigue and muscle soreness scores increased across the recovery period in both conditions. These results are consistent with previous research demonstrating an increase in perceptual fatigue after a soccer match play [20]. However, HI, fatigue and muscle soreness scores were lower in the HYP compared to the NORM condition in our study. These results are consistent with Sperlich et al. [2], who reported that HYP improved the recovery of ratings of perceived exertion following high-intensity exercise compared to the NORM condition. This finding is of high importance since the improvement of the perception of recovery and the reduction of muscle pain would have a positive effect on players’ wellbeing and physical performance, as suggested elsewhere [30, 31]. The potential mechanism of the ergogenic effect of hyperoxia could be associated with the muscle afferent activation pattern or brain oxygenation. Regarding the first hypothesis, it has been shown that the accumulation of metabolic by-products increases III and IV muscle afferent activation, leading to greater peripheral fatigue [32]. Interestingly, increasing the O_2_ availability would attenuate the accumulation of fatigue-related metabolites, and this would reduce the group III and IV muscle afferent activation. In addition, it has been shown that hyperoxic gas breathing can maintain cerebral oxygenation [1, 16]. These afore-mentioned changes could have contributed to the improvement in the wellbeing and physical performance of the players during the recovery period in our study.

While this study demonstrated the benefits of short-term, 15-minute HYP application after exercise, the potential effects of alternative doses or extended durations remain unexplored. Chronic exposure to hyperoxia, depending on its duration and intensity, can lead to significant health issues due to cellular damage or dysfunction caused by increased production of reactive oxygen species (ROS) [33]. Therefore, its use requires careful consideration, if at all. However, HYP recovery during training at moderate altitude has not been associated with elevated oxidative stress compared to training in ambient air [34]. Similarly, endurance athletes engaging in interval training with HYP recovery showed no significant increase in blood markers of oxidative stress [35]. Furthermore, the timing of HYP application may influence its efficacy. For instance, post-training HYP could facilitate recovery between sessions, potentially improving training adaptations, whereas post-match HYP, as used in the present study, appears particularly effective for recovery from acute, high-intensity efforts. Findings from Huang et al. [36] indicate that the phase of application (pre-, post-, or intra-exercise) significantly impacts outcomes, with intra-exercise use showing promising effects on muscle endurance. Future research should examine these variables to establish optimal protocols tailored to specific recovery needs. This study has certain limitations. Firstly, the muscle and cerebral oxygenation levels were not assessed. Using near infrared spectroscopy (NIRS) would have allowed the evaluation of the brain and muscle oxygenation and would have helped in understanding the potential mechanisms. Secondly, MVC is not a direct measure of football performance, and this information should be evaluated by the practitioners.

## CONCLUSIONS

The administration of HYP for 15 minutes immediately after a simulated soccer match, and daily for three subsequent days, attenuated the decline in MVC and reduced perceptual fatigue, muscle soreness, and HI scores compared to NORM conditions in semi-professional soccer players. These beneficial effects were observed as early as 15 minutes after exercise and persisted for up to 48 hours during the recovery period. The results highlight the potential of short-term HYP interventions to accelerate recovery following simulated soccer match play. Future research should explore the effects of varying HYP dosages and timing strategies, such as post-training versus post-match application, to further optimize recovery protocols for athletic populations.

## Practical applications

From a practical perspective, incorporating sessions of hyperoxia breathing to speed up perceptual recovery and the recovery of muscle strength seems both beneficial and practical. This approach could be relevant during a congested fixture schedule. One of the great advantages is that hyperoxia breathing is a low-cost and time-efficient method of recovery.
